# Proteomics and Expression of HIF2α/BNIP3L Signaling in Yak Brains at Different Altitudes

**DOI:** 10.3390/ijms26041675

**Published:** 2025-02-16

**Authors:** Qian Zhang, Yan Cui, Sijiu Yu, Junfeng He, Yangyang Pan, Meng Wang, Jialing Che

**Affiliations:** 1 College of Veterinary Medicine, Gansu Agricultural University, Lanzhou 730070, China; zq880204@126.com (Q.Z.); hejf@gsau.edu.cn (J.H.); wangmeng@gsau.edu.cn (M.W.); cj13101212713@126.com (J.C.); 2 Gansu Province Livestock Embryo Engineering Research Center, College of Veterinary Medicine, Gansu Agricultural University, Lanzhou 730070, China; sjiyu@163.com (S.Y.); panyangyang_2007@126.com (Y.P.)

**Keywords:** HIF2α, BNIP3L, LC3-II, Beclin1, cleaved caspase-3, brain, yak

## Abstract

The yak, a unique inhabitant of low-oxygen environments, exhibits brain adaptability to hypoxic conditions. However, the impact of hypoxia on yak brain proteomics and the expression of the HIF2α/BNIP3L signaling pathway remains unexplored. This study utilized TMT-based proteomics analysis to identify differentially expressed proteins (DEPs) in the cerebral cortexes of 9-month-old yaks at high (*n* = 3) and low (*n* = 3) altitudes. Additionally, qRT-PCR, Western blot, immunohistochemistry, and immunofluorescence were used to analyze HIF2α, BNIP3L, Beclin1, LC3-II, and cleaved caspase-3 expression in various brain regions from both altitude groups. KEGG analysis revealed that the DEPs were mainly concentrated in the synthesis and metabolism, DNA replication, and repair pathways. Specifically, the autophagy in KEGG attracted our attention due to its absence in other animals. HIF2α, BNIP3L, Beclin1, and LC3-II in the autophagy pathway increased significantly. Furthermore, the results of qRT-PCR and Western blot analysis showed that, at the same altitude, the mRNA and protein levels of HIF2α, BNIP3L, LC3-II, and Beclin1 in the cerebral cortexes and hippocampi of yaks were significantly higher than those in the thalami, medulla oblongatae, and cerebella (*p* < 0.05), while the expression of cleaved caspase-3 was not significantly different among the regions (*p* > 0.05). Additionally, within the same brain region, the expression levels of HIF2α, BNIP3L, Beclin1, and LC3-II in high-altitude yaks were higher than those in low-altitude yaks. Moreover, there was no difference in the cleaved caspase-3 mRNA and protein expression between the high-altitude and low-altitude yaks. Immunohistochemistry revealed that HIF2α-positive signaling was expressed in the nucleus and cytoplasm of neurons, while BNIP3L, LC3-II, Beclin1, and cleaved caspase-3 were concentrated in the cytoplasm. The immunofluorescence results showed that HIF2α, BNIP3L, LC3-II, Beclin1, cleaved caspase-3, and NeuN were co-located in the neurons of the cerebral cortex, hippocampus, thalamus, medulla oblongata, and cerebellum, respectively. This study offers a complete characterization of the yak cerebral cortex proteome at different altitudes. The higher expression of HIF2α, BNIP3L, Beclin1, and LC3-II in the cerebral cortexes and hippocampi of yaks indicates that these brain regions are more resistant to hypoxia. In addition, the increased HIF2α/BNIP3L signaling in the high-altitude yaks may enhance brain tissue adaptation to hypoxic conditions.

## 1. Introduction

The mammalian brain is organized into distinct anatomical regions, each consisting of specialized neuronal circuits. Brain function is characterized by dendritic length and density, synapse formation, glial activity, and metabolic activity. Neurons require enough oxygen to maintain homeostasis and support interneuron connectivity, synaptic activity, and cognitive function [1]. Wilson et al. showed that the mammalian central nervous system is extremely sensitive to oxygen [2]. Moreover, hypoxia and ischemia are most commonly responsible for acute plateau brain damage, leading to serious neurological diseases by causing neuronal apoptosis [3].

The yak (*Bos grunniens*) lives in plateau areas at an altitude of 2500–6000 m. Low oxygen levels are one of the most significant environmental characteristics of plateaus [4,5]. Through long-term natural selection, the native yak has formed a unique adaptive structure and physiological mechanism in these environments. In recent years, studies on the adaptation of yaks to low-oxygen environments have mostly focused on the urinary, cardiovascular, respiratory, and digestive systems [6,7,8,9]. Additionally, some studies have confirmed that yak brain tissue has developed a unique adaptation mechanism that ensures successful survival in a hypoxic environment. The number of layers and types of neurons in the cerebral cortex of a yak are similar to those of other mammals [10,11]. No proteomic studies on the cerebral cortexes of yaks at different altitudes have been reported thus far.

In the brain, autophagy is primarily studied in neurons, where its induction plays a neuroprotective role, preventing neuronal apoptosis and ensuring neuron health and survival [12,13]. HIF2α/BNIP3L are required for the induction of autophagy in hypoxia. HIF2α (hypoxia-inducible factor 2α) is a key regulator of cellular responses to low oxygen. Its activation enables cells to survive in hypoxic conditions [14]. Additionally, HIF2α can induce autophagy in neurons via the BNIP3L pathway (BCL2/adenovirus E1B 19 kDa interacting protein 3-like). In hypoxia, BNIP3L mRNA and protein levels can be up-regulated, specifically because of the hypoxia response element motif in the BNIP3L promoter region [15]. BNIP3L is located on the outer mitochondrial membrane, where it recruits the precursors to autophagosomes in order to up-regulate Beclin1 (Bcl-2 homologous domain protein), and LC3-II (microtubule-associated protein 1 light chain 3-II) throughout the brain [16]. Generally, the activation of hypoxia-induced autophagy clears damaged mitochondria, inhibiting the activation of caspase 3 and downstream apoptosis pathways [17]. It is speculated that HIF2α/BNIP3L autophagy signaling-related factors play an important role in the hypoxic adaptability of yak brains. However, there are currently no investigations of the expression of HIF2α/BNIP3L signaling-related factors in the yak brain.

In this study, TMT-based proteomics analysis was used to identify differentially expressed proteins (DEPs) in the brains of 9-month-old yaks at high and low altitudes. Moreover, qRT-PCR, Western blot, immunohistochemistry, and immunofluorescence were used to identify the expression levels of the HIF2α/BNIP3L signaling-related factors involved in the autophagy pathway of the yak brain. This study contributes new knowledge to the proteomics of cerebral cortexes in yaks from different altitudes. Furthermore, it also provides valuable information on the function and mechanism of HIF2α/BNIP3L in the yak brain, thereby laying the foundation for understanding hypoxia adaptation in the brain.

## 2. Results

### 2.1. Protein Expression in the Cerebral Cortex of High-Altitude and Low-Altitude Yaks

**Figure 1:**
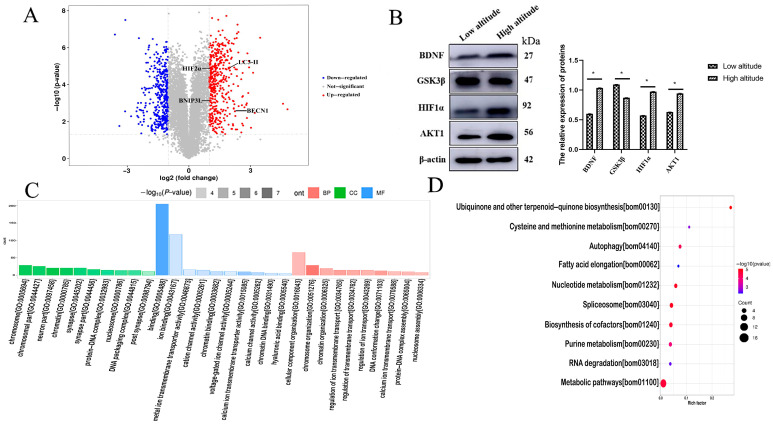
(**A**) Volcano map of differentially expressed proteins in the yak cerebral cortexes at different altitudes (high altitude vs. low altitude). The left side of the dotted line represents significantly down-regulated proteins, and the right side of the dotted line represents significantly up-regulated proteins. (**B**) The protein bands and relative expression of 4 DEPs in the cerebral cortexes of high- and low-altitude yaks. * *p* < 0.05. (**C**) The top 30 GO items in the DEP enrichment analysis of the cerebral cortex in yaks at different altitudes (high altitude vs. low altitude). (**D**) The top 10 KEGG pathways in the enrichment analysis of DEPs in the cerebral cortexes of yaks at different altitudes (high altitude vs. low altitude).

The results of the quantitative protein principal component analysis for all groups are presented in the graph below (Figure S1). In total, 856 differentially expressed proteins (DEPs) were found in the cerebral cortexes of yaks from a high altitude versus those from a low altitude (Table S1). Among them, 429 proteins were down-regulated and 427 proteins were up-regulated (Figure 1A). Four DEPs were randomly selected for Western blot verification (Figure 1B), and the results are consistent with those of the TMT analysis.

According to the GO enrichment results, 238 GO terms belonged to the biological process (BP) category, 83 GO terms belonged to the cellular component (CC) category, and 82 GO terms belonged to the molecular function (MF) category (Table S2). The top 10 significantly enriched terms are shown in Figure 1C. The enriched BP terms included cellular components, chromosome organization, and chromatin organization. The proteins that were enriched in the MF category included binding, ion binding, and metal ion transmembrane transporter activity. In the CC category, the DEPs were mainly clustered in the chromosomes, chromosome parts, and neuron parts.

Up to 14 pathways were identified through the KEGG analysis. These DEPs were concentrated in metabolic pathways, spliceosomes, cofactor biosynthesis, nucleotide metabolism, cysteine and methionine metabolism, purine metabolism, RNA degradation, autophagy, fatty acid elongation, and ubiquinone and other terpenoid–quinone biosynthesis (Figure 1D). Based on the above results, these pathways were mainly involved in synthesis and metabolism, as well as DNA replication and repair. We specifically focused on the autophagy pathway, where the levels of HIF2α, BNIP3L, Beclin1, and LC3-II increased significantly.

### 2.2. HIF2α, BNIP3L, LC3-II, Beclin1, and Caspase-3 mRNA Expression in the Brain Tissues of Yaks

**Figure 2:**
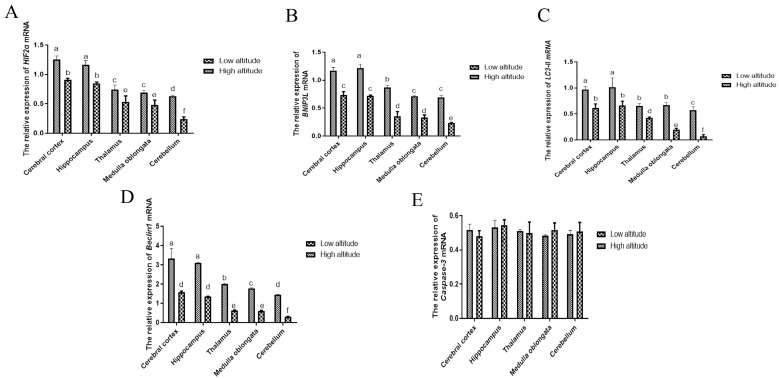
The gene expression levels of HIF2α (**A**), BNIP3L (**B**), LC3-II (**C**), Beclin1 (**D**), and caspase-3 (**E**) in the cerebral cortexes, hippocampi, thalami, medulla oblongatae, and cerebella of high- and low-altitude yaks. The gene expression levels represent the mRNA levels in relation to the mRNA expression of the control gene (β-actin). The data are expressed as the means ± SE of 2^−ΔΔCt^. Bars with different superscripts are significantly different (*p* < 0.05).

The qRT-PCR results show that, at the same altitude, the mRNA levels of HIF2α, BNIP3L, Beclin1, and LC3-II in the brain tissues of yaks were the highest in the cerebral cortex and hippocampus, and significantly higher than those in the thalamus, medulla oblongata, and cerebellum (*p* < 0.05) (Figure 2). Moreover, it was found that the HIF2α, BNIP3L, Beclin1, and LC3-II mRNA expression levels were almost significantly lower in the cerebral cortexes, hippocampi, thalami, medulla oblongatae, and cerebella of the low-altitude yaks compared to the high-altitude ones (*p* < 0.05).

The qRT-PCR results indicated that there were no significant differences in the expression of caspase-3 mRNA among the cerebral cortex, hippocampus, thalamus, medulla oblongata, and cerebellum in either the low-altitude or high-altitude yaks (*p* > 0.05). Intriguingly, the expression levels of caspase-3 in the high-altitude yaks were consistently not different from those in the low-altitude yaks tested (*p* < 0.05) (Figure 2).

### 2.3. HIF2α, BNIP3L, LC3-II, Beclin1, and Cleaved Caspase-3 Protein Expression in the Brain Tissues of Yaks

**Figure 3:**
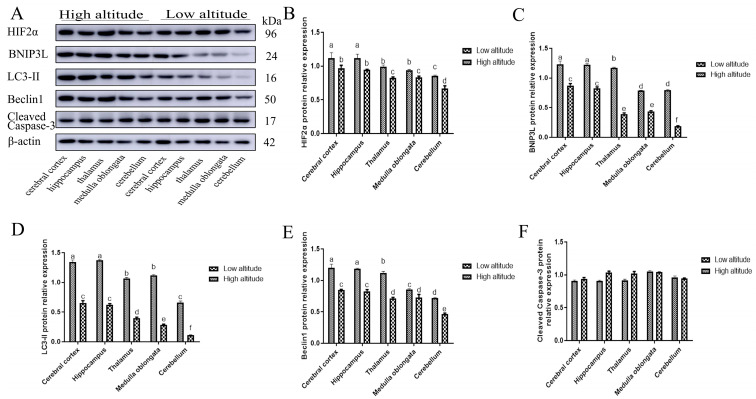
(**A**) The Western blot results. (**B**–**F**) The relative expression levels of HIF2α (**B**), BNIP3L (**C**), LC3-II (**D**), Beclin1 (**E**), and cleaved caspase-3 (**F**) in the cerebral cortexes, hippocampi, thalami, medulla oblongatae, and cerebella of high- and low-altitude yaks. The values indicate the mean ± SE. Bars with different superscripts are significantly different (*p* < 0.05).

**Figure 4:**
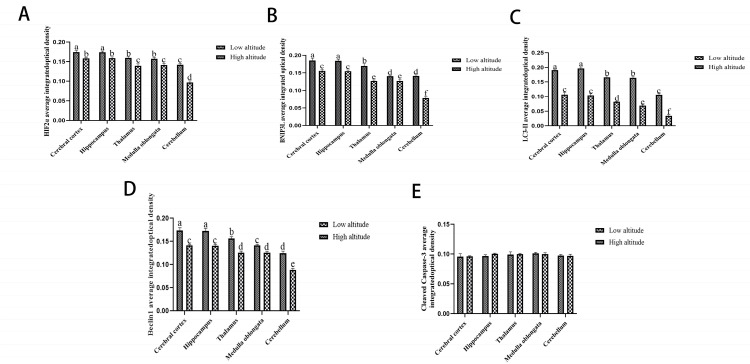
The IHC optical density analysis values of HIF2α (**A**), BNIP3L (**B**), LC3-II (**C**), Beclin1 (**D**), and cleaved caspase-3 (**E**) in the cerebral cortexes, hippocampi, thalami, medulla oblongatae, and cerebella of high- and low-altitude yaks. The data are expressed as the mean ± SE. Bars with different superscripts are significantly different (*p* < 0.05).

The expression levels of the HIF2α, BNIP3L, LC3-II, and Beclin1 proteins were determined using Western blot and immunohistochemical intensity analysis (Figure 3 and Figure 4). Within each altitude group, the expression levels of the HIF2α, BNIP3L, LC3-II, and Beclin1 proteins were higher in the yak cerebral cortex and hippocampus, followed by the thalamus, medulla oblongata, and cerebellum (*p* < 0.05). Additionally, it was observed that the protein expression levels of HIF2α, BNIP3L, LC3-II, and Beclin1 were lower in the cerebral cortexes, hippocampi, thalami, medulla oblongatae, and cerebella of low-altitude yaks than in those at high altitudes (*p* < 0.05).

In yaks at different altitudes, there were no significant differences in the cleavedcaspase-3 protein expression among the cerebral cortex, hippocampus, thalamus, medulla oblongata, and cerebellum (*p* > 0.05). Furthermore, no consistent differences in the cleaved caspase-3 protein levels were observed between the low-altitude and high-altitude yaks in these regions (*p* < 0.05).

### 2.4. HIF2α, BNIP3L, LC3-II, Beclin1, and Cleaved Caspase-3 Protein Distribution in the Brain Tissues of Yaks

**Figure 5:**
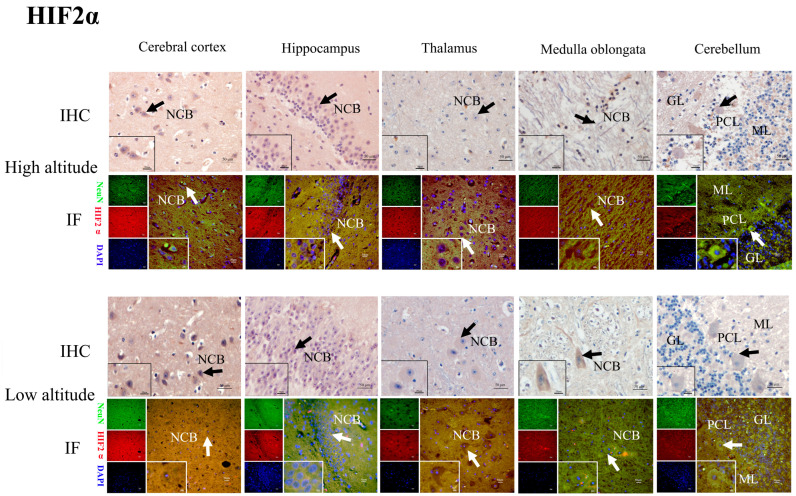
Immunohistochemical (IHC) and immunofluorescence (IF) staining of HIF2α in the cerebral cortexes, hippocampi, thalami, medulla oblongatae, and cerebella of high- and low-altitude yaks. Arrowheads indicate examples of positive cells. Abbreviations: NCB, neuronal cell body; GL, granular layer; PCL, Purkinje cell layer; ML, molecular layer. Bar = 50 μm (low-power lens, ×400); bar = 20 μm (high-power lens on the lower left, ×1000). NeuN-positive particles showed green fluorescence under the microscope, while HIF2α-positive particles showed red fluorescence. DAPI showed blue fluorescence. The same applies to the captions for Figure 6, Figure 7, Figure 8 and Figure 9.

**Figure 6:**
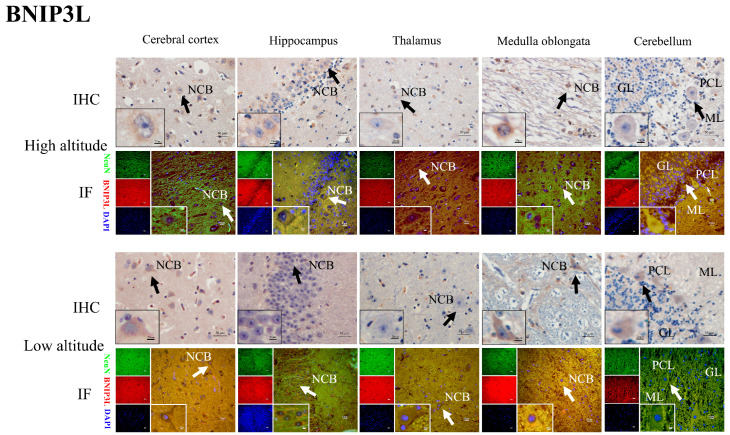
IHC and IF staining of BNIP3L in the cerebral cortexes, hippocampi, thalami, medulla oblongatae, and cerebella of high- and low-altitude yaks. NeuN-positive particles showed green fluorescence under the microscope, while BNIP3L-positive particles showed red fluorescence. DAPI showed blue fluorescence.

**Figure 7:**
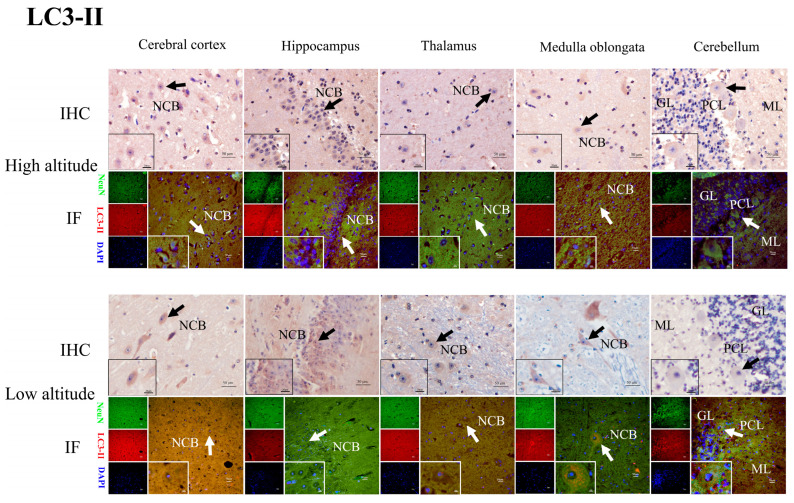
IHC and IF staining of LC3-II in the cerebral cortexes, hippocampi, thalami, medulla oblongatae, and cerebella of high- and low-altitude yaks. NeuN-positive particles showed green fluorescence under the microscope, while LC3-II-positive particles showed red fluorescence. DAPI showed blue fluorescence.

**Figure 8:**
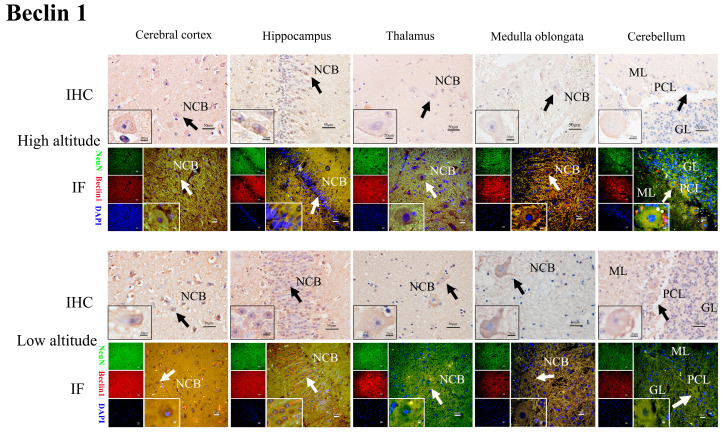
IHC and IF staining of Beclin1 in the cerebral cortexes, hippocampi, thalami, medulla oblongatae, and cerebella of high- and low-altitude yaks. NeuN-positive particles showed green fluorescence under the microscope, while Beclin1-positive particles showed red fluorescence. DAPI showed blue fluorescence.

**Figure 9:**
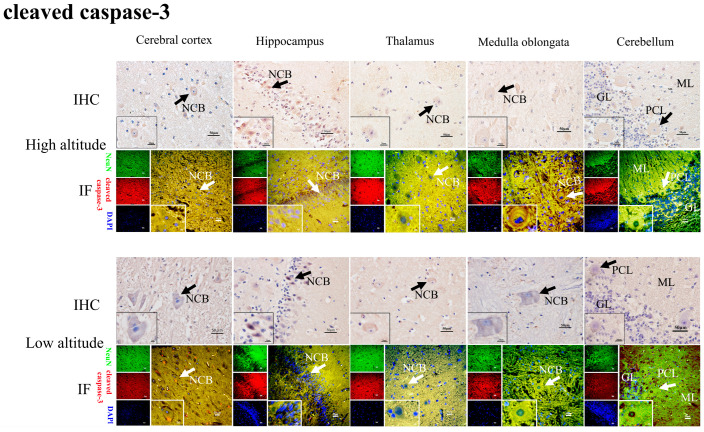
IHC and IF staining of cleaved caspase-3 in the cerebral cortexes, hippocampi, thalami, medulla oblongatae, and cerebella of high- and low-altitude yaks. NeuN-positive particles showed green fluorescence under the microscope, while cleaved caspase-3-positive particles showed red fluorescence. DAPI showed blue fluorescence.

The immunohistochemical results show that the positive reactions of HIF2α (Figure 5), BNIP3L (Figure 6), LC3-II (Figure 7), Beclin1 (Figure 8), and cleaved caspase-3 (Figure 9) were distributed in the cytoplasm of neurons, mainly in the polymorphic cell layer of the cerebral cortex, the pyramidal cell layer of the hippocampal CA region, the thalamus, the medulla oblongata, and the cerebellum. Additionally, the positive expression of HIF2α was observed in the nuclei of neurons (Figure 5).

### 2.5. Immunofluorescence Analysis

The results of the immunofluorescence analysis showed that the fluorescence expression locations of HIF2α (Figure 5), BNIP3L (Figure 6), LC3-II (Figure 7), Beclin1 (Figure 8), and cleaved caspase-3 (Figure 9) were basically the same as those of the positive immunohistochemical products, and they were all expressed in the cellular bodies of neurons. The positive staining was mostly distributed in the pyramidal-layer neurons of the cerebral cortex, the granular-cell-layer neurons of the hippocampus, the cellular bodies of the thalamus and medulla oblongata, and the Purkinje cells and a few granular cells in the cerebellum. When double-immunofluorescence staining with the NeuN antibody, it was found that the fluorescence positions of the above five factors after the merge coincided with the NeuN expression positions shown by yellow fluorescence.

## 3. Discussion

Proteomics provides the potential to identify changes in protein levels following hypoxic exposure, and it may provide important insights into the mechanisms involved in the adaptation of the yak cerebral cortex. The proteomic expression profile of the cerebral cortexes of high- and low-altitude yaks was analyzed, and 856 DEPs were screened, including 427 up-regulated DEPs and 429 down-regulated DEPs. The results of the GO analysis show that most of the DEPs were clustered in the chromosome, chromosome part, neuron part, binding, ion binding, metal ion transmembrane transporter activity, cellular component, chromosome organization, and chromatin organization categories. Similarly, Zheng et al. reported a GO analysis showing that most DEPs between hypoxic and normoxic rats were enriched in the metabolism, protein synthesis and turnover, electron transport, signaling transduction, stress response, transporter, and cell cycle categories [18]. In this study, most of the DEPs of yaks were closely related to the processes of energy production, transcription, and translation, indicating that protein synthesis, energy utilization, and neuronal activity play an important regulatory role in the adaptation of the yak cerebral cortex at different altitudes.

Cui et al. reported that the main functions of DEPs between normal and hypoxic mouse brains were energy metabolism, transfer proteins, and oxygen transportation [19]. Similarly, the KEGG analysis revealed that most of the DEPs between yaks from different altitudes were mainly clustered in metabolic pathways, spliceosomes, cofactor biosynthesis, nucleotide metabolism, cysteine and methionine metabolism, purine metabolism, RNA degradation, autophagy, fatty acid elongation, and ubiquinone and other types of terpenoid–quinone biosynthesis. These pathways are closely associated with metabolic processes, transcription, and translation. Therefore, it was speculated that metabolism plays a crucial role in the cerebral cortexes of yaks at different altitudes. Notably, the autophagy pathway in KEGG attracted our attention due to its absence in studies involving mice and rats [18,19]. In addition, proteins associated with the autophagy pathway were found to be up-regulated in the brains of high-altitude yaks compared to those at lower altitudes.

Autophagy is activated to degrade damaged organelles, proteins, and metabolic waste, thereby preventing mitochondria-dependent apoptosis in ischemic neurons [20,21]. Recent studies have demonstrated that the level of autophagy markers in hypoxic neurons is regulated by HIF2α, which is up-regulated during hypoxia [16,22,23]. In addition, it was reported that HIF2α was also effective at prolonging hypoxia by activating the expression of target genes involved in angiogenesis, cell growth, apoptosis, energy metabolism, and vasomotor regulation and thus facilitated cell survival under conditions of oxygen deprivation in Tibetan pigs [24]. In this study, we observed that in the same-altitude groups, the expression levels of HIF2α mRNA and protein were higher in the cerebral cortexes and hippocampi of yaks, followed by the thalami, medulla oblongatae, and cerebella. The regional accumulation of HIF2α protein in yak brain tissue reflects the different susceptibility of brain tissues to hypoxia. It was speculated that the cerebral cortexes and hippocampi of yaks might be the most susceptible regions to hypoxia.

Novak et al. showed that the BH3-only protein BNIP3L is an autophagy receptor that signals the autophagic degradation of mitochondria (mitophagy) via the interaction of its LC3-interacting region [25]. Additionally, recent research showed that brain damage was more serious in BNIP3L −/− mice, while more neurons survived after BNIP3L up-regulation in a cerebral ischemia injury [26]. In our study, within the same-altitude groups, the BNIP3L mRNA and protein expression levels were higher in the hippocampi and cerebral cortexes compared to the thalami, medulla oblongatae, and cerebella of yaks. Moreover, the expression level of BNIP3L was consistent with the expression patterns of HIF2α mRNA and protein in yak brains. Bellot et al. showed that BNIP3L, as a target gene directly regulated by HIF2α transcription, is an important signal of hypoxia-induced mitochondrial autophagy and that it protects against hypoxia brain injury [16]. It has been suggested that HIF2α might regulate the expression of BNIP3L in the hippocampi and cerebral cortexes of yaks.

Beclin1 is an autophagy-related protein that is necessary for autophagosome formation. In addition, it has clearly been demonstrated that the silencing of Beclin1, a major actor and initiator of autophagy, enhances cell death in hypoxia [26]. Moreover, LC3-II is an important autophagy marker, as it is a constituent of the autophagosome membrane. Lu et al. identified both increased LC3-II protein levels and autophagosome formation in a hippocampal slice culture exposed to hypoxic conditions [27]. Similarly, within the same-altitude groups, we observed that Beclin1 and LC3-II were expressed in five regions of the yak brain. Moreover, Marinković et al. and Mazure et al. demonstrated that the typical BH3 domain of BNIP3L can compete with the Beclin1–Bcl-2 and Beclin1–Bcl-XL complexes, releasing Beclin1 from the complex and then enhancing LC3-II to mediate mitochondrial autophagy [28,29]. In this study, we documented that the expression of Beclin1 and LC3-II in the cerebral cortexes and hippocampi of yaks were higher than those in the thalami, medulla oblongatae, and cerebella. These expression patterns are consistent with the characteristic expression patterns of HIF2α and BNIP3L. This implies that HIF2α and BNIP3L are highly expressed in the cerebral cortex and hippocampus, which may help regulate the expression of Beclin1 and LC3-II more effectively than in the thalamus, medulla oblongata, and cerebellum.

Neuronal apoptosis is an important change during hypoxia. Autophagy is a mechanism that protects cells from apoptosis responding to hypoxic stress. Ma et al. observed apoptosis over a certain period of time after an ischemic brain injury by TUNEL staining and found that the apoptosis rate and number of degenerative neurons increased significantly after an ischemic brain injury. However, when the BNIP3L protein level was up-regulated, the apoptosis rate and number of degenerative neurons decreased [23]. Zhang et al. and Li et al. reported that BNIP3L-related mitophagy occurred earlier than BNIP3L-mediated apoptosis and that BNIP3L-related mitophagy might be more powerful than the other form of apoptosis [30,31]. Cleaved caspase-3 is a major activator of apoptosis. In the study, at the same altitude, the cleaved caspase-3 mRNA and protein levels in the cerebral cortexes and hippocampi were not significantly different from those in the thalami, medulla oblongatae, and cerebella of yaks. This may be related to the protective effect of HIF2α/BNIP3L signaling-mediated autophagy. In other words, the higher expression of HIF2α, BNIP3L, Beclin1, and LC3-II in the cerebral cortexes and hippocampi of yaks might play a protective role in the neurons by inhibiting apoptosis.

In the same brain tissue, the expression levels of HIF2α, BNIP3L, Beclin1, and LC3-II were higher in high-altitude yaks than in low-altitude yaks. Similarly, Cai et al. reported that HIF2α was different in the brains of Zokors (genus Myospalax) at low and high altitudes [32]. Interestingly, the expression levels of cleaved caspase-3 mRNA and protein in high-altitude yaks were not significantly different from those in low-altitude yaks. It was speculated that as the altitude increased, the reduced oxygen content resulted in elevated HIF2α, BNIP3L, Beclin1, and LC3-II mRNA and protein levels in the brain tissues of high-altitude yaks. These increases may lead to a reduction in cleaved caspase-3 expression and enhance the tolerance in the brain tissues of high-altitude yaks.

Additionally, immunohistochemical and immunofluorescence staining showed that HIF2α was positively expressed in the nucleus and cytoplasm of neurons in the yak brain tissues. The positive product distribution of BNIP3L, Beclin1, LC3-II, and cleaved caspase-3 was only found in the neuronal cytoplasm. Moreover, the activation of HIF2α as a transcription factor may regulate its target gene, BNIP3L, which is involved in hypoxia-induced mitophagy throughout the brain, promoting an adaptive response by decreasing cellular apoptosis [16,33,34]. Notably, the nuclear localization of HIF2α potentially regulates the expression of BNIP3L, Beclin1, LC3-II, and cleaved caspase-3, facilitating the hypoxic adaptation of yak brain tissues.

## 4. Materials and Methods

### 4.1. Tissue Collection

All the experimental animals were treated in accordance with the Animal Ethics Procedures and Guidelines of the People’s Republic of China (approval number: 2006-398), and this study received approval from the Institutional Animal Care and Use Committee at the College of Veterinary Medicine, Gansu Agricultural University (approval number: GAU-LC-2020-32). Healthy yaks were selected based on the findings of a physical examination and serum biochemical analysis results. The animals were humanely euthanized through an intravenous administration of pentobarbital sodium (180 mg/kg).

Brain tissues were collected from 9-month-old female yaks (*n* = 3 + 3) residing at altitudes of 2500 m or 4500 m in the Qinghai area. The body weight of each yak was approximately 83 kg. The frontal lobe of the cerebral cortex, hippocampus, thalamus, medulla oblongata, and cerebellar hemispheres were excised from each animal and fixed in 4% neutral paraformaldehyde in a phosphate buffer (pH of 7.2) for subsequent immunohistochemistry and immunofluorescence detection. Samples for TMT-based proteomics analysis, real-time quantitative polymerase chain reaction (qRT-PCR), and Western blotting were preserved in liquid nitrogen until further processing.

### 4.2. Antibodies

Polyclonal rabbit anti-HIF2α (Bioss, Beijing, China; bs-1447R), polyclonal rabbit anti-BNIP3L (abcam, Cambridge, UK; ab155010), polyclonal rabbit anti-LC3-II (Abcam, Cambridge, UK; ab48394), polyclonal rabbit anti-cleaved caspase-3 (Affinity, Cincinnati, OH, USA; AF7022), polyclonal rabbit anti-BDNF (Bioss, Beijing, China; bs-4989R), polyclonal rabbit anti-GSK3β (Bioss, Beijing, China; bs-4079R), polyclonal rabbit anti-AKT1 (Bioss, Beijing, China; bs-0115R), polyclonal rabbit anti-HIF1α (Bioss, Beijing, China; bs-0737R), polyclonal rabbit anti-Beclin1 (Bioss, Beijing, China; bs-1353R), monoclonal mouse anti-NeuN (Invitrogen, Carlsbad, CA, USA; MA5-33103), and polyclonal rabbit anti-β-actin (Bioss, Beijing, China; bs-0061R) antibodies were used. The primary antibodies used in Western blot, IHC, and IF were the same.

### 4.3. Protein Extraction and Trypsin Digestion

Cerebral cortex samples were homogenized in a lysis buffer containing 8 M urea, 1% protease inhibitor, and 1% phosphatase inhibitor, followed by sonication. Subsequently, the mixture was clarified by centrifugation at 20,000× *g* for 10 min at 4 °C to remove any residual debris. The clear supernatant was then aliquoted into a fresh tube. The protein concentration was subsequently determined using a BCA protein assay kit (Abcam, Cambridge, UK).

Dithiothreitol (Sigma, Shanghai, China) was introduced to the protein sample to achieve a final concentration of 5 mM, followed by incubation at 56 °C for 30 min. Subsequently, iodoacetamide (Sigma, Livonia, MI, USA) was incorporated at a concentration of 11 mM. The sample was then incubated at ambient temperature in darkness for 15 min. The urea concentration was reduced to 2 M by incrementally adding 100 mM triethylammonium bicarbonate (TEAB; Sigma, Shanghai, China) to the protein solution. Lastly, the sample underwent overnight digestion at 37 °C with trypsin (at a ratio of 1:50 trypsin to protein; Promega, Madison, WI, USA), followed by a second digestion for 4 h at a 1:100 trypsin-to-protein ratio.

### 4.4. Quantitative Protein Mass Spectrometry TMT Labeling

Peptides resulting from trypsin digestion were purified using Strata X C18 columns (Phenomenex, Torrance, CA, USA) and subsequently subjected to vacuum freeze-drying. For reconstitution, these peptides were dissolved in 0.5 M TEAB, following the protocol provided with the TMT labeling kit (Thermo Fisher Scientific, Waltham, MA, USA).

### 4.5. Classification

The peptides underwent fractional distillation via high-pH reverse-phase HPLC, utilizing an Agilent 300 Extend C18 column with the specifications of a 5 μm particle size, a 4.6 mm internal diameter, and a 250 mm length. In summary, the peptides were eluted to yield 60 fractions across a gradient of 8–32% acetonitrile at pH 9.0, over a duration exceeding 60 min. These fractions were subsequently consolidated into six major groups and dehydrated using a vacuum centrifugation process.

### 4.6. LC-MS/MS Analysis

The tryptic peptides were prepared in a mixture containing 0.1% formic acid and 2% acetonitrile, referred to as solvent A, and then analyzed using an EASY-nLC 1200 Ultra-Performance Liquid Chromatography system. The complementary solvent, solvent B, was composed of 0.1% formic acid and 90% acetonitrile. The elution gradient was configured to start at 6% and linearly increased to 22% within the initial 38 min. It then increased to 32% over the subsequent 14 min, escalated to 80% in the next 4 min, and was maintained at 80% for the final 4 min, all at a steady flow rate of 450 nL/min.

In ultraperformance liquid chromatography (UPLC) analysis, peptides were introduced via a nanospray ionization (NSI) source and detected on a Q Exactive HF-X mass spectrometer (Thermo) with an electrospray voltage of 2.0 kV. The peptides underwent a full scan across a mass-to-charge (*m*/*z*) range of 350 to 1600 and were further characterized using an Orbitrap detector set at resolutions of 120,000 for proteomics analyses. The MS/MS settings were adjusted to an *m*/*z* range of 100 with resolutions of 15,000 for proteomics. Data acquisition was performed using a data-dependent acquisition (DDA) method.

### 4.7. Proteomics Data Analysis

Experimental secondary mass spectrometry data were analyzed using Proteome Discoverer (version 2.4.0.305). A search was conducted against the uniprot-Bos mutus_72004-2021-7.fasta database, which was augmented with inverse libraries to estimate the false discovery rate (FDR) from random matches and with common contamination libraries to eliminate contaminating proteins from the identification results.

The enzymatic cleavage method was set to Trypsin (Full), allowing up to two missed cleavage sites. The minimum peptide length was six amino acid residues, with a maximum of three modifications per peptide. The mass error tolerance was set at 10 ppm for primary parent ions and 0.02 Da for secondary fragment ions. Fixed modifications included Carbamidomethyl ©, TMT6plex (peptide N-Terminus), and TMT6plex (K), while variable modifications included Acetyl (protein N-Terminus) and Oxidation (M). The quantification method was TMT-10 plex, with a false discovery rate (FDR) set at 1% for protein, peptide, and PSM identification. The identification accuracy FDR was set at 1% for the spectral, peptide, and protein levels.

Differentially expressed proteins were identified based on the two-fold change (fold change <0.5 or >2) and a *p*-value of <0.05. Protein functions were annotated using Gene Ontology, and enriched pathways were analyzed using the Kyoto Encyclopedia of Genes and Genomes (KEGG) database. Both GO and KEGG enrichment analyses employed a two-tailed Fisher’s exact test.

### 4.8. Real-Time Quantitative Polymerase Chain Reaction (RT-qPCR)

TRIzol reagent (Thermo Fisher Scientific, Waltham, MA, USA) was utilized to extract the total RNA from frozen tissue samples. The Evo M-MLV Reverse Transcription Kit (Accurate Biology, Changsha, China) was used to reverse-transcribe RNA into cDNA, following the manufacturer’s instructions. The resulting cDNA products were stored at −80 °C for RT-qPCR analysis. The primers for RT-qPCR were designed based on Bos grunniens sequences using Primer 5 software and synthesized at the Beijing Genomics Institute (BGI, Shenzhen, China) (Table 1). A real-time PCR detection system (Light Cycler VR 96; Roche, Basel, Switzerland) was employed for RT-qPCR, with a reaction volume of 20 μL that contained 1.5 μL of cDNA (400 ng/μL), 0.75 μL of forward and reverse primers (0.2 μmol/mL), 10 μL of SYBR Green II master mix (Takara, Dalian, China), and 7 μL of nuclease-free H_2_O. The RT-qPCR conditions were as follows: one cycle at 95 °C for 3 min, followed by 40 cycles at 95 °C for 10 s, 60 °C for 30 s, and 72 °C for 20 s. Each sample was analyzed in four replicates, with Bos grunniens β-actin serving as an internal control. The relative changes in gene expression were then calculated using the relative quantification method 2^−∆∆Ct^.

### 4.9. Western Blot Assays

The frozen samples were used to extract the total proteins by employing an extraction buffer (Beyotime, Shanghai, China), and the proteins were subjected to denaturation at 100 °C for a duration of 8 min. An enhanced BCA protein assay kit (Thermo Fisher Scientific, Niederelbert, Germany) was utilized to quantify all the proteins. Subsequently, equal amounts of protein (20 μg) were separated using 10% SDS-polyacrylamide gel electrophoresis and transferred onto polyvinylidene fluoride membranes (Amersham, Burlington, Massachusetts, USA). To block the membranes, they were treated with a solution containing 5% skimmed milk in Tris-buffered saline supplemented with 0.1% Tween 20 at room temperature for approximately 1.5 h. Following this step, primary antibodies, including anti-BDNF (1:400 dilution), anti-GSK3β (1:800 dilution), anti-HIF1α (1:600 dilution), anti-AKT1 (1:1000 dilution), anti-HIF2α (1:400 dilution), anti-BNIP3L (1:800 dilution), anti-LC3-II (1:1000 dilution), anti-Beclin1 (1:600 dilution), and anti-cleaved caspase-3 (1:1000 dilution), were incubated overnight at 4 °C. The anti-β-actin antibody was employed as the loading control using a concentration ratio of 1:1000. The membranes underwent five washes with phosphate-buffered saline containing 0.01% Tween 20, followed by an incubation period of 2 h with the goat anti-rabbit IgM antibody (Bioss, Beijing, China, bs-0295G-HRP, 1:2000 dilution). After another round of washing five times, the bands on the membranes were visualized utilizing an enhanced chemiluminescence reagent. ImageJ software (verion: 1.5.4) was employed to calculate the relative expression levels of the BDNF, GSK3β, HIF1α, AKT1, HIF2α, BNIP3L, Beclin1, LC3-II, and cleaved caspase-3 proteins based on the band densities obtained from the analysis.

### 4.10. Immunohistochemical Examination

An immunohistochemical staining technique was utilized to assess the spatial distribution of HIF2α-, BNIP3L-, LC3-II-, Beclin1-, and cleaved caspase-3-positive cells in yak brains. Paraffin blocks were prepared and 4 μm thick sections were obtained. Following standard procedures, the fixed tissue specimens were mounted on microscope slides and incubated with primary antibodies against HIF2α (1:50 dilution), BNIP3L (1:50 dilution), LC3-II (1:50 dilution), Beclin1 (1:100 dilution), and cleaved caspase-3 (1:200 dilution) for 2 h at a temperature of 37 °C within a moist chamber. A biotinylated anti-rabbit secondary antibody (SP-0023, Bioss, Beijing, China) was applied for 20 min. Subsequently, the slides were treated with streptavidin-conjugated peroxidase for 10 min. Positive products were formed with 3,3-diaminobenzidine tetrahydrochloride (c-0010, Bioss, Beijing, China). The sections were lightly stained with hematoxylin as a counterstain. The negative control had the primary antibody replaced with rabbit serum albumin, with all the other steps and conditions remaining the same. The intensity in the immunohistochemistry was measured using integrated optical density analysis and Image-Pro plus 6.0.

### 4.11. Double Immunofluorescent Staining

The methods of immunofluorescence tissue embedding, sectioning, dewaxing, and antigen repair were the same as those of the immunohistochemistry procedure. Slices were incubated overnight at 4 °C with the primary antibodies anti-HIF2α (1:50 dilution), BNIP3L (1:50 dilution), LC3-II (1:50 dilution), Beclin1 (1:100 dilution), cleaved caspase-3 (1:200 dilution), and mouse anti-NeuN (1:400, Millipore, Bedford, MA, USA). After incubation with secondary antibodies (mouse anti-rabbit IgG (H&L)/AF594, bs-0295M-AF594; rabbit anti-mouse IgM/AF488, bs-0368R-AF488, Bioss, Beijing, China), 4,6-diamidino-2-phenylindole dihydrochloride (DAPI) was used to stain the cell nuclei. Finally, the sections were observed with a Leica fluorescence microscope (Leica, Wetzlar, Germany). The negative control was incubated with PBS instead of the primary antibody. NeuN-positive particles showed green fluorescence under the microscope, and the positive particles with other factors showed red fluorescence. The fluorescence was observed after the two overlapped in yellow.

### 4.12. Statistical Analysis

All the data are presented as the means ± standard errors. The statistical analysis was performed using IBM SPSS (version 26.0; SPSS Inc., Chicago, IL, USA). Comparisons were analyzed using one-way ANOVA. The animal was considered a random effect, and the location was considered a fixed effect. *p* < 0.05 was regarded as statistically significant.

## 5. Conclusions

This work makes an important contribution toward defining the proteomic differences in the cerebral cortexes of yaks living at different altitudes. Within the same-altitude groups, the expression levels of HIF2α, BNIP3L, Beclin1, and LC3-II were higher in the hippocampi and cerebral cortexes of the yaks. Conversely, there were no significant differences in the protein expression of cleaved caspase-3 among the cerebral cortex, hippocampus, thalamus, medulla oblongata, and cerebellum. This suggests that the higher expression level of HIF2α, BNIP3L, Beclin1, and LC3-II in the cerebral cortex and hippocampus potentially protect against apoptosis. Furthermore, the expression levels of HIF2α/BNIP3L signaling-related factors were higher in the brain tissues of high-altitude yaks than in those of the low-altitude yaks, possibly indicating a role in adaption. These findings provide valuable information for studying the hypoxic adaptive mechanism of the plateau yak brain.

## Figures and Tables

**Table 1:** Primer sequences.

Gene	Sequence	Annealing Temperature (°C)	Size (bp)
HIF2α	F: ACCGGGCAAGTGAGAGTC		
	R: GATGTCCATGTGGGATGGGT	60	123
BNIP3L	F: CTCGGCTTGTTGTGTTGCTG		
	R: GACTGTTCGCCTTCCTCACA	54	151
Caspase-3	F: AACTGGACTGTGGTATTGAGAC		
	R: AGCCTGTGAGCGTACTTATTC	55	190
LC3-II	F: CCGACTTATCCGAGAGCAGC		
	R: TGAGCTGTAAGCGCCTTCTT	56	161
Beclin1	F: GGCTGAGGCTGAGAGGTTGGAT R: CATCTGGGCATAACGCATCTGGTT	59	130
β-actin	F: GCAATGAGCGGTTCC		
	R: CCGTGTTGGCGTAGAG	60	141

## Data Availability

All data generated during the current study are included in this manuscript.

## Supplementary Materials

The following supporting information can be downloaded at https://www.mdpi.com/article/10.3390/ijms26041675/s1.
