# Supplementary figures and images for: Proteomics and Expression of HIF2α/BNIP3L Signaling in Yak Brains at Different Altitudes
Source: Int J Mol Sci. 2025 Feb 16;26(4):1675. doi: 10.3390/ijms26041675 (PMC11855096; doi:10.3390/ijms26041675)

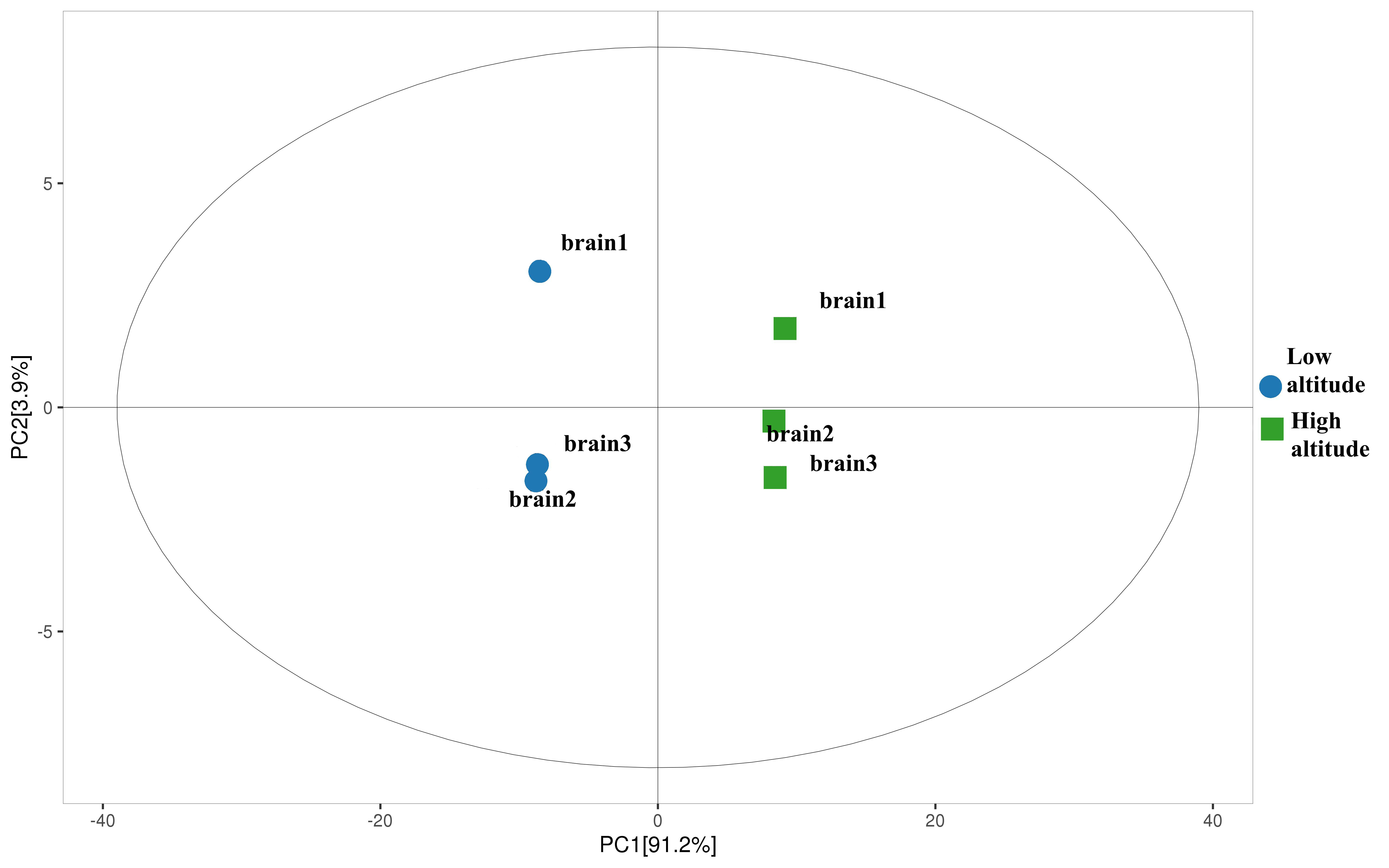

Supplement: Supplementary file 1 [file ijms-26-01675-s001.zip › Figure S1.jpg]
